# Mutation Analysis of *BRAF*, *MEK1* and *MEK2* in 15 Ovarian Cancer Cell Lines: Implications for Therapy

**DOI:** 10.1371/journal.pone.0001279

**Published:** 2007-12-05

**Authors:** Anne L. Estep, Chana Palmer, Frank McCormick, Katherine A. Rauen

**Affiliations:** 1 Comprehensive Cancer Center and Cancer Research Institute, University of California at San Francisco, San Francisco, California, United States of America; 2 Canary Foundation, San Jose, California, United States of America; 3 Department of Microbiology and Immunology, University of California at San Francisco, San Francisco, California, United States of America; 4 Department of Pediatrics, University of California at San Francisco, San Francisco, California, United States of America; Ohio State University Medical Center, United States of America

## Abstract

**Background:**

Among gynecologic cancers, ovarian cancer is the second most common and has the highest death rate. Cancer is a genetic disorder and arises due to the accumulation of somatic mutations in critical genes. An understanding of the genetic basis of ovarian cancer has implications both for early detection and for therapeutic intervention in this population of patients.

**Methodology/Principal Findings:**

Fifteen ovarian cancer cell lines, commonly used for in vitro experiments, were screened for mutations using bidirectional direct sequencing in all coding regions of *BRAF*, *MEK1* and *MEK2*. *BRAF* mutations were identified in four of the fifteen ovarian cancer cell lines studied. Together, these four cell lines contained four different *BRAF* mutations, two of which were novel. ES-2 had the common B-Raf p.V600E mutation in exon 15 and Hey contained an exon 11 missense mutation, p.G464E. The two novel B-Raf mutants identified were a 5 amino acid heterozygous deletion p.N486-P490del in OV90, and an exon 4 missense substitution p.Q201H in OVCAR 10. One of the cell lines, ES-2, contained a mutation in MEK1, specifically, a novel heterozygous missense substitution, p.D67N which resulted from a nt 199 G→A transition. None of the cell lines contained coding region mutations in *MEK2*. Functional characterization of the MEK1 mutant p.D67N by transient transfection with subsequent Western blot analysis demonstrated increased ERK phosphorylation as compared to controls.

**Conclusions/Significance:**

In this study, we report novel *BRAF* mutations in exon 4 and exon 12 and also report the first mutation in *MEK1* associated with human cancer. Functional data indicate the *MEK1* mutation may confer alteration of activation through the MAPK pathway. The significance of these findings is that *BRAF* and *MEK1/2* mutations may be more common than anticipated in ovarian cancer which could have important implications for treatment of patients with this disease and suggests potential new therapeutic avenues.

## Introduction

Ovarian cancer is the second most common gynecologic cancer in the United States affecting approximately 22,000 women each year causing an estimated 15,200 deaths [1]. Cancer is a genetic disorder arising from the accumulation of somatic mutations in genes involved in critical cellular pathways. These mutations typically result in proteins which exhibit their oncogenic effect by altering signaling through vital transduction networks, or in haploinsufficiency of critical tumor suppressor proteins.

Understanding the genetic basis of ovarian cancer has implications both for early detection, as well as for therapeutic intervention in this population of patients. Genes which have been found somatically mutated in ovarian cancer include *KRAS* [2]–[5], *NRAS* [2], *PIK3CA* [5]–[9], *PTEN* [5], [10], [11], *TP53* [5], [12], [13] and *BRAF* [2]–[4]. B-Raf, the protein product of *BRAF*, is a serine/threonine protein kinase and the first in the mitogen-activated protein kinase (MAPK) cascade which is one of the many downstream effector pathways of Ras. Extracellular stimuli leads to the activation of Ras, which in turn activates Raf (A-Raf, B-Raf, and/or C-Raf-1). Raf then phosphorylates and activates MEK1 and/or MEK2 (MAPK kinase). MEK1 and MEK2 are threonine/tyrosine kinases with both isoforms having the ability to phosphorylate and activate ERK1 and ERK2 (MAPK). ERK, once activated by MEK, has numerous cytosolic and nuclear substrates [14]. Aberrant upstream signaling resulting in hyperactivated ERK plays a key role in the pathogenesis and progression of approximately 30% of human cancers, including ovarian cancer [15]. As a result, this pathway has been an attractive target for the development of small molecule inhibitors for the treatment of cancer [16].

Genes comprising the MAPK pathway, *BRAF*, *MEK1* and *MEK2*, were systematically scanned for mutations in 15 ovarian cancer cell lines using bidirectional direct sequencing of all exons. Previous reports have demonstrated that B-Raf is mutated in approximately 28–37% of low grade serous carcinomas [3], [17]. With this information, our search was expanded to include *MEK1* and *MEK2*, two genes along with *BRAF*, which we have recently determined to be causal for cardio-facio-cutaneous (CFC) syndrome (MIM 115150), a multiple congenital anomaly syndrome whereby individuals have characteristic craniofacial dysmorphia, heart defects and ectodermal anomalies [18]. Interestingly, unlike germline mutations identified in CFC syndrome, no somatic mutations have ever been identified in *MEK1/2* in any cancer type. In our analysis, novel *BRAF* mutations were identified in exon 4 and exon 12 of two separate cell lines. In addition, we report the first functional mutation in *MEK1* associated with human cancer. The significance of these findings is that *BRAF* and *MEK1/2* mutations may be more common than previously recognized in ovarian cancer, which could have important implications for the treatment of patients with ovarian cancer.

## Results

### BRAF and MEK1 Mutations

**Figure 1:**
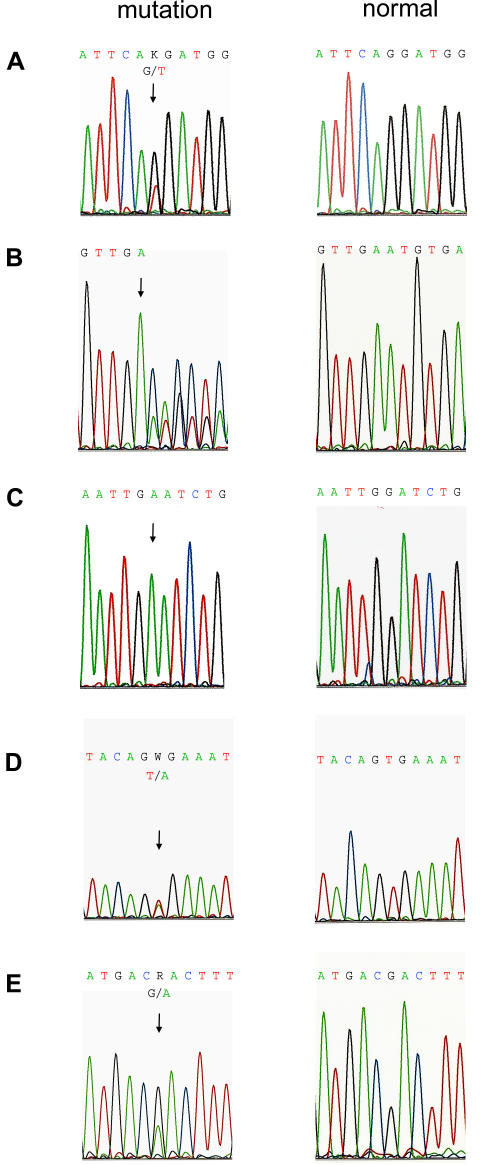
Electropherograms of *BRAF* and *MEK1* mutations compared to normal controls. Four *BRAF* mutations were identified in four individual cell lines. A) OVCAR 10 contained a nt 603 G→T transversion causing a heterozygous missense substitution p.Q201H in exon 4. B) OV90 contained a novel heterozygous deletion starting at nt 1457 (arrow) resulting in a 5 amino acid deletion, p.N486-P490del, in exon 12. C) Hey contained a nt 1391 G→A transition resulting in loss of heterozygosity. D) ES-2 contained an exon 15, T→A transversion at nt 1799, substituting glutamic acid for valine at position 600 (p.V600E). E) A nt 199 G→A transition in *MEK1,* exon 2 resulted in a heterozygous missense substitution, p.D67N.

Genomic DNA from 15 ovarian cancer cell lines was screened for *BRAF* mutations in coding exons 1–18. Four mutations were identified in four individual cell lines: OVCAR 10, OV90, Hey and ES-2 (Figure 1). None of the other cell lines had *BRAF* mutations. Two of the four *BRAF* mutations identified were novel. OVCAR 10 contained a nt 603 G→T transversion causing a heterozygous missense substitution p.Q201H in exon 4 (Figure 1A). OV90 contained a novel heterozygous 5 amino acid deletion, p.N486-P490del, in exon 12 (Figure 1B). In addition to the two novel *BRAF* mutations identified, two additional mutations which have been previously reported in cancer were identified. Hey contained a nt 1391 G→A transition resulting in an exon 11 missense mutation, p.G464E (Figure 1C). The electropherogram demonstrated loss of heterozygosity at this locus. ES-2 contained an exon 15, T→A transversion at nt 1799, substituting glutamic acid for valine at position 600 (p.V600E) (Figure 1D). None of these mutations were identified in the controls.

All eleven coding exons of *MEK1* and *MEK2* were also sequenced in the same cell lines and controls. One mutation in *MEK1* was identified in ES-2 consisting of a novel heterozygous missense substitution, p.D67N, which resulted from a nt 199 G→A transition (Figure 1E). No other nonsynonymous substitutions in MEK1 were identified. All eleven coding exons of *MEK2* were sequenced and no nonsynonymous substitutions were identified.

### Nucleotide Variation

In addition to these mutations, a total of four different synonymous single nucleotide polymorphisms (SNPs) were identified in *BRAF* (Table 1), *MEK1* (Table 2), and *MEK2* (Table 3). Three of these four SNPs were found in five or more of the fifteen cell lines and have been previously reported (www.ncbi.nlm.nih.gov/SNP/). In order of frequency, the three synonymous database SNPs include: i) MEK2 p.I220I (rs10250) present in 11 of the 15 cell lines (73%), ii) BRAF p.G634G (rs9648696) found in six of the 15 cell lines (40%) and, iii) MEK2 p.D151D (rs17851657) identified in five of the 15 cell lines (33%). There was one uniquely identified MEK1 SNP in OVCAR 10 resulting from a nt 348 heterozygous G→A transition in exon 3, p.Q113Q (Table 2). All synonymous SNPs were characterized by SIFT (Sorting Intolerant From Tolerant) and determined to be tolerated.

**Table 1:** *BRAF* sequence variations identified in ovarian cancer cell lines.

Cell Line	Exon	Nucleotide Mutation	Amino Acid Mutation	dbSNP*	Predicted Protein Function**
OVCAR 10	4	603 G>GT	p.Q201H	N/A	tolerated
Hey	11	1391 G>AA	p.G464E	N/A	affected
OV90	12	Δ1457-1471	p.N486-P490 del	N/A	affected
ES-2	15	1799 T>TA	p.V600E	N/A	affected
A2780	16	1929 A>AG	p.G643G	rs9648696	tolerated
OV90	16	1929 A>AG	p.G643G	rs9648696	tolerated
A1847	16	1929 A>AG	p.G643G	rs9648696	tolerated
IGROV 1	16	1929 A>AG	p.G643G	rs9648696	tolerated
2008	16	1929 A>GG	p.G643G	rs9648696	tolerated
OVCAR 10	16	1929 A>GG	p.G643G	rs9648696	tolerated

* www.ncbi.nlm.nih.gov/SNP/

** blocks.fhcrc.org/sift/SIFT.html

**Table 2:** *MEK1* sequence variations identified in ovarian cancer cell lines.

Cell Line	Exon	Nucleotide Mutation	Amino Acid Mutation	dbSNP*	Predicted Protein Function **
ES-2	2	199 G>GA	p.D67N	N/A	affected
OVCAR 10	3	348 G>GA	p.Q116Q	none	tolerated

* www.ncbi.nlm.nih.gov/SNP/

** blocks.fhcrc.org/sift/SIFT.html

**Table 3:** *MEK2* synonymous SNPs identified in ovarian cancer cell lines.

Cell Line	Exon	Nucleotide Mutation	Amino Acid Mutation	dbSNP*	Predicted Protein Function **
OVCAR 3	4	453 C>CT	p.D151D	rs17851657	tolerated
SKOV 3	4	453 C>CT	p.D151D	rs17851657	tolerated
ES-2	4	453 C>CT	p.D151D	rs17851657	tolerated
2008	4	453 C>CT	p.D151D	rs17851657	tolerated
Hey	4	453 C>CT	p.D151D	rs17851657	tolerated
OVCAR 3	6	660 A>AC	p.I220I	rs10250	tolerated
SKOV 3	6	660 A>CC	p.I220I	rs10250	tolerated
A2780	6	660 A>AC	p.I220I	rs10250	tolerated
OV90	6	660 A>CC	p.I220I	rs10250	tolerated
ES-2	6	660 A>CC	p.I220I	rs10250	tolerated
TOV-21g	6	660 A>CC	p.I220I	rs10250	tolerated
OVCAR 5	6	660 A>CC	p.I220I	rs10250	tolerated
2008	6	660 A>AC	p.I220I	rs10250	tolerated
OVCAR 10	6	660 A>AC	p.I220I	rs10250	tolerated
PEO1	6	660 A>AC	p.I220I	rs10250	tolerated
Hey	6	660 A>AC	p.I220I	rs10250	tolerated

* www.ncbi.nlm.nih.gov/SNP/

** blocks.fhcrc.org/sift/SIFT.html

### Functional Characterization of Mutants

SIFT was utilized to characterize the functional significance of the nonsynonymous amino acid substitutions identified in B-Raf and MEK1. B-Raf p.G464E, p.N486-P490del, p.V600E and MEK1 p.D67N were predicted to be deleterious substitutions causing an alteration of protein function, whereas B-Raf p.Q201H was predicted to be tolerated.

**Figure 2:**
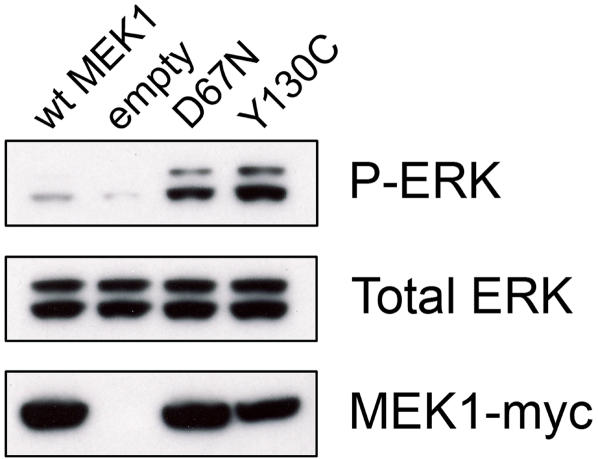
Functional characterization of the MEK1 p.D67N mutant identified in ES-2. Human embryonic kidney 293T cells were transiently transfected with empty vector, wild-type MEK1, MEK1 p.Y130C (positive control mutant which has known high activity level[18]) and the MEK1 p.D67N mutant. ERK (p44 ERK1 and p42 ERK2) phosphorylation was assayed by Western blotting using phospho-specific antibodies. The p.D67N MEK1 mutant had increased ERK phosphorylation compared to the level induced by empty vector and wildtype MEK1. The level of ERK phosphorylation induced by p.D67N MEK1 is slightly less than the CFC MEK1 p.Y130C mutant which is known to have increased activity [18]. Myc-tagged MEK1 is shown for transfection efficiency and total ERK is shown as a loading control.

To corroborate the functional alteration of the novel MEK1 p.D67N mutant identified in ES-2, transient transfection of HEK 293T cells with subsequent Western blot analysis demonstrated increased kinase activity as measured by ERK phosphorylation (Figure 2). The MEK1 p.D67N mutant had increased ERK phosphorylation compared to wildtype MEK1. The level of ERK phosphorylation was lower than the CFC MEK1 p.Y130C mutant which is known to have a high level of activity (positive control).

## Discussion

Altered signaling through the MAPK pathway in cancer often results from mutations in upstream components of ERK, including K-Ras, N-Ras, H-Ras, C-Raf-1 and B-Raf [15]. Molecular studies of ovarian cancer cell lines and tumor specimens have identified genetic mutations in some of these genes, *KRAS*, *NRAS* and *BRAF* , which result in the alteration of signaling through this critical pathway [2], [19]–[23].

Somatic mutations in *BRAF* have been reported at a high frequency in numerous cancers including melanoma, thyroid, colorectal and ovarian. Approximately 70 missense mutations affecting 34 codons have been reported (www.sanger.ac.uk/genetics/CGP/cosmic). In cancer, the majority of somatic *BRAF* mutations result in missense substitutions found in, but not limited to, exon 11 (the glycine-rich loop) and exon 15 (the activation segment) in the protein kinase domain [24]. One missense mutation in exon 15 resulting in a missense substitution, B-Raf p.V600E, accounts for over 90% of *BRAF* mutations identified in human cancer. The crystal structure of B-Raf shows that the activation segment is held in an inactive conformation by association with the G-loop. Mutations in these two regions, the glycine-rich loop and the activation segment, are believed to disrupt this interaction, converting B-Raf into its active conformation [25].

In addition to somatic mutations, germline mutations in *BRAF* have recently been identified as causing CFC syndrome, a multiple congenital anomaly disorder whereby individuals have characteristic craniofacial dysmorphisms, cardiac defects, ectodermal anomalies and developmental delay [18], [26]. Most of the mutations in CFC syndrome fall outside the exon 11 and exon 15 protein kinase domain. The most common causative CFC mutation, p.Q257R, resides in exon 6 of the cysteine-rich domain. Like most cancer-causing mutations in *BRAF*, biochemical studies have determined that most novel CFC B-Raf mutant proteins have increased kinase activity relative to the kinase activity of wild-type B-Raf [18], [26]. These studies punctuate the fact that *BRAF* mutations occur not only somatically but also in the germline, and that mutations that confer increased kinase activity are not restricted to those typically associated with cancer.

Bidirectional sequencing of all the coding exons of *BRAF* in 15 well-characterized and highly utilized ovarian cancer cell lines identified four cell lines with *BRAF* mutations. Only one cell line had the common exon 15 *BRAF* mutation. ES-2 which is derived from ovarian clear cell carcinoma [27] had the common heterozygous p.V600E missense mutation. Previous reports have demonstrated that B-Raf is mutated most commonly in low grade ovarian serous carcinomas with a frequency of approximately 28–37% and that all mutations are the common B-Raf p.V600E variant [3], [17]. Interestingly, the two serous-derived cell lines examined in this study (Hey and OV90) did not have the common B-Raf p.V600E mutation; however, both did contain *BRAF* mutations. Hey, derived from a papillary serous carcinoma [28], contained an exon 11 missense mutation, p.G464E. This missense mutation has been described previously (www.sanger.ac.uk/genetics/CGP/cosmic) and is also a codon which is mutated in CFC syndrome ([29]; Rauen, unpublished data). OV90 which is derived from a serous adenocarcinoma [30] contained a novel mutation in exon 12 resulting in a heterozygous five amino acid deletion, p.N486-P490del. Although extremely rare, somatic exon 15 B-Raf in-frame deletion-insertions have been reported in cancer [31], [32]. In addition, we identified two CFC individuals with small in-frame deletions in exon 11 (Rauen, unpublished data). Finally, OVCAR 10, which is derived from a human ovarian epithelial cancer, had a unique heterozygous missense substitution p.Q201H in exon 4. This nonsynonymous SNP has not been identified in cancer or CFC syndrome and was determined to be tolerated by SIFT, so perhaps B-Raf p.Q210H represents a rare polymorphism.

Only two of the ovarian cancer cell lines had mutations in the typically affected exon 11/15 regions in the B-Raf protein kinase domain. This finding raises the possibility that cancer-associated *BRAF* mutations outside of exon 11/15 may be more common than anticipated. Since the vast majority of *BRAF* mutation survey studies published are restricted to exons 11 and 15, other possible mutations outside these regions may be overlooked.

To explore the functional significance of these novel B-Raf mutants, all those identified in this study were analyzed by SIFT (http://blocks.fhcrc.org/sift/SIFT.html), an online mutation analysis program that predicts the functional consequence of nonsynonymous amino acid substitutions [33], [34]. All *BRAF* mutations identified were predicted to have altered protein function except for p.Q201H. The functional significance of *BRAF* coding mutations in exons other than 11 and 15 is supported by biochemical studies of novel germline mutations identified in CFC. Like somatic mutations, most CFC germline mutations confer an increased kinase activity, while some are kinase impaired. However, all CFC mutations which have been biochemically characterized alter protein function to some extent ([18], [26]; Rauen, unpublished data).

B-Raf has only two known downstream effectors, MEK1 and MEK2. In an effort to characterize the mutation spectrum of the MAPK pathway in ovarian cancer, we also sequenced *MEK1/2* and identified a novel MEK1 heterozygous missense substitution, p.D67N, in ES-2 which resulted from a nt 199 G→A transition. This is the first identified functional MEK mutation associated with cancer and does not represent a rare polymorphism in that this mutation was not identified in 40 normal controls (80 alleles) or in 52 CFC individuals (104 alleles) we have sequenced to date ( [18]; Rauen, unpublished data). Interestingly, although we had not identified this MEK1 p.D67N mutant in our CFC cohort, this same MEK1 mutation has recently been reported as a germline mutation in CFC syndrome [29]. In addition to a *MEK1* mutation, ES-2 also had a B-Raf p.V600E missense substitution. These two mutations may have been co-operating tumorigenic events. What makes this finding particularly interesting is the fact that previous sequencing studies of the MEK1/2 protein kinase domain failed to identify any mutations in gliomas, testicular germ cell tumors, breast cancer and lung cancer [35]–[38]. Notably, the p.D67N in ES-2 falls outside the protein kinase domain which spans from AA 68-271 (www.ensembl.org/Homo_sapiens). Many germline CFC mutations are located 5’ of the protein kinase domain ([18], [29], [39]; Rauen, unpublished data). Functional studies of these novel CFC mutants have demonstrated increased activity *in vitro* over wildtype MEK in stimulating ERK phosphorylation, but these CFC mutants are not as active as an artificially generated constitutively active MEK mutant ([18]; Rauen, unpublished data). Other studies have demonstrated that alteration of the N-terminus of MEK increases the basal kinase activity implicating an important regulatory role along with substrate recognition [40]–[42].

To assess the functional consequence of MEK1 p.D67N, this amino acid substitution was analyzed by SIFT and found to be functionally affected. To corroborate this information, MEK1 p.D67N was transiently transfected into HEK 293T cells, and ERK phosphorylation was measured by Western blot analysis. The MEK1 p.D67N mutant is activating as demonstrated by increased ERK phosphorylation as compared to empty vector and wildtype MEK1, two controls which do not activate ERK. However, the level of ERK phosphorylation for p.D67N is less than the positive control CFC MEK1 p.Y130C mutant [18].

In addition to *BRAF* and *MEK1* mutations, several synonymous database SNP were also identified. B-Raf p.G634G (rs9648696) was identified in 40% of the 15 cell lines, MEK2 p.I220I (rs10250) was present in 73% and MEK2 p.D151D (rs17851657) was identified in 33% of the cell lines. These synonymous database SNPs were present at a frequency which is comparable to that previously reported (http://www.ncbi.nlm.nih.gov/SNP/snp; [43]). There was one uniquely identified *MEK1* SNP in OVCAR 10 resulting from a nt 348 heterozygous G→A transition in exon 3, p.Q113Q. All synonymous SNPs were characterized by SIFT and determined to be tolerated.

Our findings emphasize that mutations which alter function of the MAPK pathway play an important role in ovarian cancer [15]. In addition, the identification of mutations in key MAPK pathway components will be important in determining the responsiveness of the cancer to therapeutics, the aggressive and metastatic behavior of the tumor and the prognosis of the patient. Four of 15 (26%) cell lines in this study had *BRAF* mutations and 1 of 15 (7%) had a MEK mutation. It is known that *BRAF* mutations have been identified in certain types of ovarian cancer; however, mutations in the downstream effectors of B-Raf including *MEK1* and *MEK2* may also be important contributors of ovarian cancer tumorigenesis. Defining the pathogenetics of ovarian cancer may enable the use of targeted therapeutics, such as small molecule inhibitors of MAPK pathway, which have recently begun to demonstrate great promise [16]. In addition, a report indicates that cells with activated B-Raf have enhanced, selective sensitivity to MEK inhibitors [44]. Our results underscore the importance that further characterization of the sensitivity of various BRAF and MEK mutants to small molecule inhibition is an important avenue to pursue towards the development of effective treatments for ovarian cancer.

## Methods

### Cell Lines and Isolation of Genomic DNA

Fifteen ovarian cancer cell lines, commonly used for *in vitro* experiments, were screened for mutations: OVCAR 3, SKOV 3, TOV-112d, A2780, OV90, ES-2, TOV-21g, Caov-3, A1847, IGROV 1, OVCAR 5, 2008, OVCAR 10, PEO1 and Hey. Cell pellets from each of these cell lines were kindly provided by Drs. Charles Drescher and Beatrice Knudsen. Genomic DNA was isolated from cell pellets using the QIAamp DNA Tissue kit (Qiagen, Valencia, CA), according to the manufacturer’s instructions. Forty healthy human controls were included in this study. Genomic DNA was isolated from peripheral blood lymphocytes using the QIAamp DNA Blood Midi kit (Qiagen, Valencia, CA), according to the manufacturer’s instructions. Control samples were obtained under an approved institution review board from the University of California San Francisco.

### PCR and Bidirectional Sequencing

PCR primers were designed to amplify all coding exons and intronic flanking regions of *BRAF* (NM_004333.2), *MEK1* (NM_002755.2) and *MEK2* (NM_030662.2) (Table 4 and Table 5). For sequencing, the PCR primers were modified on the 5′ end to include M13 forward (GTAAAACGACGGCCAGT) and reverse (CAGGAAACAGCTATGACC) sequences. PCR and sequencing were performed by Agencourt Bioscience Corporation (Beverly, MA). Bidirectional sequencing was conducted with ABI BigDye v3.1 Cycle Sequencing Kit (Applied Biosystems, Foster City, CA) according to manufacturer’s guidelines and run on an ABI3730xl capillary sequencing instrument (Applied Biosystems, Foster City, CA).

**Table 4:** *BRAF* Sequencing Primers.

Exon	Forward	Reverse	bp
1	GCTCTCCGCCTCCCTTCC	GGCCATTGTGTGTGTTTACG	405
2	GAAGGCTGCTCACCAAACC	TCTTCCCAAATCTATTCCTAATCC	551
3	TGAAACTTAAAACCCTATCAACTGG	GATGCCTCTATTTGCATGACC	500
4	TGTAGAAATGGTGTTGTATCTGACC	CCAAATAAATCACACTCTGAATGG	515
5	TGTTACTAGCCCCTCGATAACC	GCTTACAGGTACAAGCACACG	577
6	TCTTCCTTTCACCTCTGTTTCC	AACAATCGTATGGAAGAAAAACC	589
7	TTTTAACAGTTGTTTCTGAGAATGG	CCAGGAGATCCAAAAGAAAGC	528
8	AGCAGCTTTGGCAGTATTGG	TCATCAGAGAGAAACCAGAAGC	518
9	CATTGGCAAGTGCTTCAAAA	TTGGGTTTCTCTACACATTTTTCTC	355
10	AATGAGGCCCCTTTTTGG	ATTCTGGACCAGCCTTTTCG	593
11	AGTAAGGGGATCTCTTCCTGTATCC	TGCTGTGAACAGTTTTTATTTCC	416
12	CATGGAACAAACAAGGTTGG	CATTGCATACTACTTAAAAGAATGTGG	511
13	TTTTTCTGACAACATTTTACCG	CCAGCCATTAGTTAGCATCC	417
14	GGCTTGACTGGAGTGAAAGG	AAAAGCAGGCTGTGGTATCC	506
15	GGAAAGCATCTCACCTCATCC	TGGTTTCAAAATATTCGTTTTAAGG	566
16	GAATCAGGAATGGGAAGTGG	TCTATCCTTCACGCTTACC	576
17	GAGAACCTTTGCCACAGTCC	AATTTCTAGGTGTGCCACTGC	541
18	TGCTTTCTTGTAAAGTGTGATGG	CCCGGAACAGAAAGTAAAGC	582

**Table 5:** *MEK1* and *MEK2* Sequencing Primers.

Gene	Exon	Forward	Reverse	bp
MEK1	1	GGTCCACTGAGACCGCTACC	CTGTACAGTGGCCAGGAACC	527
	2	CCTCTCTAGCCTCCCACTTTG	CAAACAGCACAAAAAGGTATTGA	452
	3	CCTGTTTCTCCTCCCTCTACC	ACACCCACCAGGAATACTGC	494
	4	AGTGCAGTGGTGTGATCTCG	CTTTCCCCTCATTGACTTCC	324
	5	GGAGGAAGGCAAATTTGTGA	GGTAGGAGTTGCTGCCTCAG	341
	6	TAACGGACTCCTTCCTGTGG	TCCTCCCTCACTTCTTGTCC	545
	7	AGGAGGCCAAATTCAAGAGG	ACAACACCCACCTGGAGACC	510
	8	TGGCTGTTTAATGTTTATTGTCC	TGGTGCTTAGTATAAAGCTGTGC	598
	9	GGATGGGGAGAGGAGATGG	ATCAGACGGGAGGGTAAAGG	252
	10	CTGTGGGCATGATACTGTGC	AACTGATGGGAGAGCAAAGG	370
	11	TTCCAAGTGCAGCACAAGC	ACACACAAATAGCCCCAAGC	461
MEK2	1	CCTGCCTCTCGGACTCG	GTGCACTCCTCGCGAACC	443
	2	CTTGAGGTCCTGAGGTCTGC	GCCTGGAGCTAATCAGAATGC	582
	3	TTGGTCTTGACCACTGTTGG	AGAAGGATCCCCTGGAAGC	380
	4	AGGCAGAACTGTCAGAAGACG	CTTGGCCACTCTCTTTCTGC	381
	5, 6	CAGCACTGTCTCGTCTCTGG	GAGAACTGGGAGGGACAGC	580
	7	GGTCATTAGCCATGGAGAGG	CACTGCTTCCAGCTCTGTCC	507
	8	CAATTTAGGCTGGCATGTGG	TGCAGCACAGTAGAAGATGG	563
	9	ATCCAGATGTCCCTCTGTGG	CTCTGGGAAAAGGAATCTGG	549
	10	CTCTCTGGTCGGAGAGATCC	TCTCATGAGGGCAAAGAAGC	490
	11	TTTGCTTTCTGTCCCGTACC	AGCTCAGGGATGTCCTCTCC	577

### Analysis

Sequencing data was analyzed using two sequence analysis programs, PolyPhred Software v5.02 (University of Washington, Seattle, WA) and SeqScape® Software (Applied Biosystems, Foster City, CA). In addition, manual review was conducted with Mutation Surveyor 3.00 (SoftGenetics LLC, State College, PA). Further evaluation of detected nucleotide mutations consisted of Sorting Intolerant From Tolerant (SIFT; blocks.fhcrc.org/sift/SIFT.html) and screening against known databases: NCBI, Cosmic, UniProtKB/Swiss-Prot and JSNP (www.ncbi.nlm.nih.gov/SNP, www.sanger.ac.uk/genetics/CGP/cosmic/, ca.expasy.org/sprot/, snp.ims.u-tokyo.ac.jp/).

### Plasmids

Human *MEK1* cDNA (Origene, Rockville, MD) was cloned into a pcDNA3 vector with a Myc-tag at the N-terminus. The *MEK1* nt 199 G→A transition was introduced using Quick-Change Site-Directed Mutagenesis (Stratagene, La Jolla, CA) and verified by direct sequencing.

### Transient Transfections and Western Blot Analysis

Human embryonic kidney (HEK) 293T cells were seeded the day before in six-well dishes and transfected, in duplicate, with 2 µg total plasmid DNA and 5 µl of Lipofectamine 2000 (Life Technologies, Carlsbad, CA) according to manufacturer’s instructions. Cells were serum-starved (0.5% fetal bovine serum) and 24 hours later lysed in buffer containing Protease and Phosphatase Inhibitor cocktails (Sigma, St. Louis, MO). Expression levels of myc-MEK, total ERK and phosphorylated ERK were analyzed by Western blot. Myc (A-14) and p-ERK(E-4) were purchased from Santa Cruz Biotechnology (Santa Cruz, CA), and p44/42 MAP Kinase antibody was purchased from Cell Signaling Technology (Danvers, MA).
